# Underrepresented populations in genomic research: a qualitative study of researchers’ perspectives

**DOI:** 10.1186/s12920-025-02140-5

**Published:** 2025-04-16

**Authors:** Arian Omeranovic, Flora Nguyen Van Long, Asma Boubaker, Annie Turgeon, Hermann Nabi

**Affiliations:** 1 Oncology Division, CHU de Québec-Université Laval Research Center, Hôpital du Saint-Sacrement, 1050, Chemin Ste-Foy, Local J0 - 01, Quebec City, QC G1S 4L8 Canada; 2 Department of Social and Preventive Medicine, Faculty of Medicine, Université Laval, 1050, Avenue de La Médecine, Quebec City, QC G1 V 0 A6 Canada

**Keywords:** Underrepresented populations, Genomic research, Population descriptors, Canada, Qualitative research, Diversity, Equity, Inclusion

## Abstract

**Background:**

The lack of diversity in genomic data limits researchers’ ability to investigate the relationships between genetic profiles, disease manifestations, and responses to new therapies. As a result, innovations in treatment could have potentially harmful effects on a significant portion of the population due to incomplete or inaccurate genomic data. In addition, the lack of harmonization in the use of population descriptors in genomic studies raises both ethical and scientific concerns regarding which descriptors should be used to study and recruit underrepresented populations. Therefore, understanding the factors contributing to the lack of diversity in genomic research is an urgent scientific, clinical, and public health priority. This study aims to explore the social and contextual factors influencing the participation of underrepresented populations in genomic research, from the perspective of researchers in the field.

**Methods:**

A total of 13 semi-structured interviews were conducted with researchers experienced in genomic research in Canada and fluent in either French or English. The interview transcripts were analyzed using thematic analysis.

**Results:**

Researchers identified several factors contributing to the low participation of underrepresented populations in genomic research, with one key factor being the geographic distribution of research institutions and the disconnect between research efforts and the communities being studied. To address this issue, participants stressed the importance of moving away from colonial practices, such as conducting research on a community without consulting its members in the design phase. Furthermore, it was suggested that existing diversity, equity, and inclusion policies alone were insufficient to effectively address the challenge. Lastly, the study also highlighted a potential link between how study populations are categorized and the willingness of underrepresented groups to participate in genomic research.

**Conclusion:**

Although researchers are generally aware of the literature on the causes, consequences, and potential solutions for increasing participation, confusion remains regarding the use of population descriptors. Our findings highlight the need for improved education, greater consensus, and expanded dialogue within the genomic research community to promote the harmonization of population descriptors.

**Supplementary Information:**

The online version contains supplementary material available at 10.1186/s12920-025-02140-5.

## Background

With the advent of precision medicine and the decreasing cost of genome sequencing [1], genome-wide association studies (GWAS) have become a cornerstone in advancing our understanding of diseases. However, 86% of GWAS samples come from individuals of European descent [2–4]. This lack of diversity in genomic data limits the discovery and understanding of associations between genetic variants, disease presentation and response to modern innovative therapies in underrepresented populations. As a result, it undermines the goal of precision medicine [5], which seeks to provide personalized care or interventions [6]. Furthermore, the underrepresentation of diverse populations is already affecting both the understanding of their health needs and the quality of the healthcare services provided [7]. In some cases, individuals from underrepresented populations have been prescribed inappropriate medication or undergone unnecessary health surveillance and interventions due to inaccurate or incomplete genomic data [8]. More broadly, this lack of diversity in genomics might exacerbate existing social inequalities in health and research [2, 9].

To address this challenge, it is crucial to understand the barriers and facilitators that contribute to the gap between populations of European descent and underrepresented groups. Documented barriers to the participation of underrepresented populations in genomic research include limited knowledge or understanding of genomics and genetic testing, concerns about confidentiality, privacy, and data governance (i.e. who has access to data, how it is managed, what it can be used for), fear of discrimination, limited access to genetic services and distrust in the healthcare system, science, and research [10–15]. From a global perspective, there is a pressing need to enhance resources and capacity building in low- and middle-income countries (LMICs) [2].

Factors that facilitate participation include personal benefits such as receiving results or monetary compensation, awareness, a good understanding of genomics, and a family history of a medical condition [10–12]. The engagement of researchers with underrepresented communities, the cultural or linguistic adaptation of research material, and community-led data governance have also been identified as key facilitators [11, 15, 16]. This engagement aligns with the widespread implementation of Diversity, Equity and Inclusion (DEI) policies in academia and the corporate world, which are often cited as solutions to correct historical biases and promote participation of underrepresented populations [17, 18]. In line with these initiatives, the three main federal research granting agencies in Canada, the Canadian Institutes of Health Research (CIHR), the Natural Sciences and Engineering Research Council of Canada (NSERC), and the Social Sciences and Humanities Research Council of Canada (SSHRC), established the Tri-Agency Statement on Equity, Diversity, and Inclusion (EDI) to “foster more equitable, diverse and inclusive research ecosystem in Canada” [19]. However, the impact of these DEI policies on Canadian genomic research is still unknown.

The lack of diversity in genomic research raises concerns about social inequalities and draws attention to the persistent issue of inconsistent use of population descriptors [20, 21]. The lack of a harmonized approach for describing and categorizing ethnic and racial groups in genomic research presents significant scientific and ethical challenges [21–24]. Notably, the confusion and lack of clear distinction between the concepts of race, ethnicity and ancestry among genomic researchers make it challenging to generalize genomic data findings from one group to another [21, 25]. For instance, as noted by Fatumo et al., the common use of the terms “African ancestry” or “Black” in genomic research fails to capture the broader ethnolinguistic and genetic diversity of the African continent, thereby contributing to inequities within an already underrepresented population [2]. Moreover, the categories used can differ not only between countries but even within different institutions in the same country, as in the United States [26]. From an ethical perspective, the inconsistency in the use of population descriptors could perpetuate racial discrimination and stigmatization of underrepresented populations [1, 20, 23], while also hindering genomic researchers’ efforts to recruit these populations. The persistent use of “race” as a population descriptor in genomics could reinforce the false notion that race is a biological reality [20, 23, 27].

However, the impact of misusing population descriptors on the participation of underrepresented groups remains unclear. While many studies have examined how genomic researchers categorize populations [21, 25, 27], none have explored the views of these researchers on the key issues contributing to the low participation of underrepresented populations. The aim of this study was to explore the social and contextual factors that influence the participation of underrepresented populations in genomic research from the perspectives of researchers in the field. To structure this investigation, the study centered on three specific topics:

1. researchers’ views on the underrepresentation of diverse populations in genomic research and identify solutions to improve their participation.
2. researchers’ practices and perspectives regarding race, ethnicity, and ancestry in genomic research.
3. the link between the use of population descriptors and the participation of underrepresented populations.

## Materials and methods

### Participants

This study was conducted with genomic researchers in Canada who were proficient in either English or French. These researchers were currently conducting or had conducted genomic research in the past five years with Canadian cohorts. The five-year timeframe was selected to include individuals who had participated in genomic research projects in the recent past. Canadian researchers were the focus for two main reasons. First, much of the existing research in this field has involved researchers from the United States [21, 25]. Despite the geographical proximity, differences in culture, research practices, and legal frameworks may influence the perspectives of genomic researchers. Second, since the research team is based in Canada, engaging researchers from Canadian institutions was more practical.

For the recruitment process, participants were selected by systematically reviewing each project currently funded by Genome Canada, a federally funded not-for-profit organization. At the time of recruitment, 68 researchers with projects based on human genomics, either partially or fully funded by Genome Canada, were selected. Although a convenience sample was used, efforts were made to recruit researchers from each Canadian province to ensure a fairly representative sample of the entire country. An email was sent to each of these researchers containing a description of the research project and a request for voluntary participation in the interview. A reminder email was sent one or two weeks after the initial invitation to increase the response rate. The time and date of the interview were scheduled once the researcher's participation was confirmed. A final email was sent containing a link to a sociodemographic questionnaire, a Zoom meeting link, and the study information and consent form, which participants were asked to sign prior to the meeting.

### Data collection

Semi-structured interviews were chosen to better understand the perspectives of genomic researchers on underrepresented populations, as this qualitative approach offers deeper insights than a questionnaire [28]. In addition, given that the topics of discrimination and racism in science may be sensitive for some participants, this data collection method is often regarded as more appropriate for exploring sensitive and complex issues [29]. Thirteen semi-structured interviews were conducted between October 2023 and March 2024. Recruitment ended after the 13 th interview, as data saturation had been reached and the target population size of ten participants was exceeded. While there is no universally established sample size for this type of data collection, it aligns with the sample sizes reported in the literature on data saturation [30, 31]. The interviews lasted an average of 30 min, ranging from 17 min to one hour. This duration aligned with the research team’s objective of conducting shorter interviews to accommodate researchers and maximize participation. Interviews were held via Zoom or Teams, except for one, which took place in Québec City. The introductory paragraph of the interview guide was read to the participant, reiterating the research theme and outlining the interview procedure, including the estimated 30-min duration and the fact that it would be audio recorded. This allowed us to reiterate that the data collected would be anonymized, as stated in the consent form. Finally, verbal consent was confirmed a second time before starting the audio recording and the first interview question.

AO conducted all interviews in French and English, depending on the participants’ language preference, using the semi-structured question guide. The interview guide was developed by the research team (A.O, A.B, H.N.) for this specific study [see Additional File 1]. Based on the relevant literature, three main topics were addressed with careful consideration of our research objective.

1. Categorization of population differences: This topic explored how researchers identified and recruited populations for their studies, as well as their perspectives and understanding of the concepts of race, ethnicity, and ancestry through open-ended questions.
2. Perspective on inequalities in genomic research: For this topic, we sought researchers'views on the causes and consequences of the underrepresentation of certain populations in genomic research.
3. Inclusion of underrepresented populations in genomic studies: This last topic was divided into three sub-topics. First, we examined researchers'opinions on diversity, equity and inclusion (DEI) policies, along with their perceived impact on research. Second, we investigated the barriers and facilitators influencing the participation of underrepresented populations in research. Finally, we examined the potential relationship between categories used in research and participation rates.

After identifying these themes, the research team (A.O, A.B, H.N.) developed open-ended questions, reaching a consensus on which to retain or remove from the final semi-structured interview guide. Finally, the guide was translated from French into English, ensuring that the core meaning of the questions remained intact.

### Data analysis

Each semi-structured interview was transcribed verbatim for thematic analysis. Using Taguette, an open-source software for qualitative data analysis [32], AO coded each response into themes and subthemes. This coding was based on the general trends in opinions and perspectives across the transcript corpus, with the most frequently mentioned themes assigned a higher position in the hierarchical categorization. This coding method is based on the work of Paillé and Mucchielli [33]. As the analyses progressed and new interview data was added, some themes were merged and subdivided into separate themes. Moreover, the themes that emerged from the interviews differed from those initially selected for the interview guide. This can be explained in part by the semi-directed nature of the data collection. While the framework guided the interview through question orientation and order, the participants could express themselves freely. In this regard, the epistemological approach is mainly inductive, with the data collected serving as the foundation for developing theories and frameworks to better understand the research topic [34]. However, it also follows a deductive approach, as the interview questions, result analysis, and research problem selection are informed by existing studies, data, and theoretical frameworks [33]. A.O, H.N. and A.B. contributed to validating the themes that emerged from the thematic analysis.

### Ethical considerations

This study was approved by the Ethics Review Boards of the CHU de Québec (Quebec City University Hospital) (#2024–6887). Each participant signed the information and consent form before participating in the interview. Since the field of genomic research in Canada has a small number of researchers, each participant was assigned a number to anonymize the extracts and prevent identification.

## Results

### Characteristics of participants and their research

Of the 68 researchers contacted, 18 agreed to participate, while six declined. Of the 18 who agreed, 13 participated in the semi-structured interviews, while the remaining five did not follow up after initially accepting the invitation. The characteristics of the participants are shown in Table 1. Most participants identified as male (69.2%) and self-identified as being of European origin (58.3%). Most had over 15 years of academic research experience (58.3%) and worked in a variety of genomic research fields such as clinical research (*n* = 5), basic research (*n* = 4), translational research (*n* = 3) and population research (*n* = 3). Regarding work locations, the majority of participants were from the provinces of Quebec (*n* = 5), British Columbia (*n* = 3), and Ontario (*n* = 2). In Alberta, New Brunswick, and Nova Scotia, one researcher from each province participated in our interviews.

**Table 1:** Participants'characteristics

	**N (%)**
**Gender**	
Female	4 (30.8)
Male	9 (69.2)
**Ethnocultural background**	
European origins	7 (58.3)
Other North American origins	3 (25.0)
African origins	2 (16.7)
Asian origins	1 (8.3)
Caribbean origins	1 (8.3)
**Location**	
Quebec	5 (38.5)
British Colombia	3 (23.1)
Ontario	2 (15.4)
Alberta	1 (7.7)
New Brunswick	1 (7.7)
Nova Scotia	1 (7.7)
**Research experience**	
Experienced [Over 15 years]	7 (58.3)
Mid-career [5 to 15 years[	3 (25.0)
Early career [0 to 5 years[	2 (16.7)
Missing	1
**Type of research**	
Clinical	5 (41.7)
Basic	4 (33.3)
Translational	3 (25.0)
Population	3 (25.0)
Other	2 (16.7)
Missing	1

Participant research themes of the participants were defined during the interviews. Most were interested in the field of oncology and equity and diversity issues in genomics. Few participants were interested in genetic profiling at the population level. Finally, one participant worked on the relationship between infectious diseases and genomics.

### Categorization and population descriptors

The research population studied by most of our interviewees was qualified as the “general population”, which refers to several different groups. Most commonly, the study population was either the Canadian population or a specific province, such as Quebecers or Albertans, with no focus on a specific ethnic group. However, some researchers mentioned populations of European origin as their main study group. Otherwise, some participants were interested in Indigenous, Acadian, and newborn populations. These populations were generally recruited by consent obtained during a clinical visit, traditional population recruitment methods such as polling firms, or through community contacts.

Following the discussion around their research population, participants also discussed the use of population descriptors in genomic research. Quotes are summarized in Table S1 (see Additional file 2). In general, participants favored the term “ancestry” to categorize populations. “Ethnicity” was the second most frequently mentioned population descriptor. As for the term “race”, it was always used in combination with either ethnicity or ancestry. Moreover, some participants preferred to use all three terms at the same time to account for the complexity of an individual origin (Quote 1, Participant #10).

When asked about using the term “race” in genomics, responses from our participants varied. Most respondents viewed race as a social construct (Quote 2, Participant #6). In addition to its social nature, many participants viewed the term as controversial or loaded and felt it required careful consideration of its use in genomics. (Quote 2–3, Participant #6–4). Nevertheless, some mentioned that the biological basis of race is reflected in the distribution of diseases and that it is important to understand genetic differences (Quote 4, Participant #12). The concept of “ancestry” was also viewed as having a biological basis. For one of our participants, this biological component differentiated ancestry from race and ethnicity (Quote 5–6, Participant #5–10). In our interviews, ethnicity was viewed as related to culture as it conveyed a more refined expression of an individual origin than other population descriptors (Quote 7, Participant #10). However, while most respondents described the social and cultural nature of ethnicity, some pointed out that ethnicity was linked to our biology (Quote 8, Participant #11).

This variety of perspectives on population descriptors was evident in our participants'difficulty distinguishing between these three concepts. Although many could differentiate race, ethnicity and ancestry, a significant proportion used the terms interchangeably or recognized the complexity of using population descriptors without providing a clear answer.

As presented in Table S1 (see Additional file 2), we also explored how researchers categorize the populations included in their studies. The most common categorization methodology/approach was self-identification of an individual's ethnicity, race or ancestry. In this approach, individuals either self-identified an origin or self-selected from a predefined list of options (Quote 9, Participant #3). This list was typically based on the categories established by Statistics Canada. Moreover, some participants reported asking about an individual’s grandparents’ origin to determine their ethnicity (Quote 10, Participant #8).

Genotyping was the second most common method of categorization reported. This approach involves using various techniques to identify genetic similarities between individuals and assigning a population descriptor or a group label based on these similarities. Participants who used biological markers to categorize their populations of interest also incorporated self-reported ethnicity, race, and/or ancestry. This combination was used to improve sampling precision or explore the relationship between individuals'self-perceptions and genetic data (Quote 11, Participant #4). For some, biological markers were also combined with various social determinants of health in their statistical models (Quote 12, Participant #10). Some participants were unable to categorize their population by ethnicity, race and/or ancestry because it was against the law for their type of research. It is important to note that no federal or provincial laws or mandates in Canada prohibit data collection on ethnicity, race, and/or ancestry in healthcare.

When discussing the usefulness of categorizing by race, ethnicity and ancestry, several participants highlighted their importance in understanding health disparities between groups, as it helps to highlight differential treatment between populations (Quote 13, Participant #6). According to one participant, the issue with current population descriptors is not in categorizing but in their misuse throughout history (Quote 14, Participant #4).

Some participants recognized that current categories lack scientific rigor, as an increasing number of individuals have diverse ethnic and ancestral backgrounds, making the boundaries between ethnic groups fluid and porous (Quote 15, Participant #11). The mixing of populations complicates categorization in genetic and scientific research (Quote 16, Participant #2). Finally, one participant cautioned against using racial and ethnic categories as substitutes for distinctions based on social phenomena (Quote 17, Participant #6).

### Relation between participation and population descriptors

The possible relation between participation and population descriptors was also explored. Quotes for this theme are available in Table 2. Participants had mixed views of racial and ethnic categorization and willingness to participate in genomic research. Few expressed strong opinions about whether categorization had negative or positive impacts. For most, categorization could have either effect since it depends on the research context (Quote 18, Participant #3). This statement highlights that nearly all of our interviewees believed that there is a relationship between participants and population descriptors, though the inner workings of the relationship are still unclear. As one participant mentioned, the proper use of population descriptors is strongly dependent on how the research is promoted and the clarity of its aims (Quote 19, Participant #1). Some participants acknowledged the complexity of individual origins. For some, this complexity was even more evident because human populations are increasingly mixed, making commonly used categories increasingly obsolete (Quote 20, Participant #2).

**Table 2:** Relation between participation and population descriptors

Subtheme	Selected quotes
Importance of context	Quote 18: *"I think it can go either way depending how it's done. So, I think if people feel that you're asking for their race and ethnicity and categorizing them in order to be able to get the best quality evidence and to be specific and to be accurate, then I think they will appreciate the chance for that. But I think if people feel that you're going to discriminate against them in any way because of it, or treat them differently, or come up with negative conclusions. Then it can go the other way."* Participant #3
	Quote 19: *"I think how research is promoted—how we do the promotion, how we make our announcements—it can go either way. I think it can be detrimental […] if we don't explain properly why we're doing it. And if we find a way of clearly explaining the aim […] [I] think that can help. If you simply mention it without explaining why, I think it can be harmful or at least give rise to fears and concerns."* Participant #1
Complexity of admixture	Quote 20: *“But the real challenge in genetics right now is admixture. For example, you can have individuals with one African ancestor and one Asian ancestor, who got married and had children. So, it’s not that simple—how will they identify themselves? […] And even people with white skin, for example. I don’t know…In France, there are a lot of people from North Africa living in France. And when couples form, how will their children identify themselves? That’s the reality nowadays, there is much more admixture."* Participant #2
Possibility in categorization	Quote 21: *"Through our community partners who are working in the hereditary cancer space. We've created posters in trying to explain the communities that we'd like to speak to. And we use the word racialized, but we've also just invited the community partners to use the words that they're comfortable with using, that their community partners and the patients and just their community members find appropriate."* Participant #5

Finally, two participants suggested solutions to mitigate the harmful effects of categorization. One proposed that population descriptors in research should include an explanatory preamble on the notions of race and ethnicity to clarify that race has no biological basis before asking about self-identification. The other participant believed the solution lies in how research is conducted, where communities are given the final say on how they wish to categorize themselves (Quote 21, Participant #5).

### Causes of diverse population underrepresentation in genomic research

Table S2 (see Additional file 3) provides an overview of our participants’ key perspectives on the causes of the underrepresentation of diverse populations in genomic research. One primary explanation highlighted by participants is the geographical disparity in research. Many pointed out that the divide between LMICs and high-income countries (HICs) disproportionately tends to benefit the latter, as most major research centers and funding are concentrated in HICs. (Quote 22–23–24, Participant #12–10 - 7). Moreover, the geographical dimension of this aspect is also evident within the HICs, where access to research is unevenly distributed between urban and rural communities (Quote 23, Participant #10). Participants noted that researchers often prioritize convenience sampling, focusing on the most accessible populations, rather than the more challenging task of reaching underrepresented populations (Quote 24, Participant #7).

Secondly, participants identified fear and mistrust regarding data governance, as well as the lasting effects of colonialism and social inequalities, as key factors contributing to underrepresentation (Quote 25, Participant #11). For instance, one of our participants highlighted that, in the Canadian context, First Nations communities are often more “concerned” and “resistant” to engaging with researchers due to past negative experiences with the scientific community. This underscores the importance of dedicating time and resources to rebuilding trustful relationships with underrepresented communities that have experienced discrimination and racism (Quote 26, Participant #13). Several participants also mentioned socio-economic inequalities as a key factor underlying the specific disparities in genomic research (Quote 27, Participant #10). One participant viewed the phenomenon as a reflection of the inequalities that permeate our society.

Thirdly, many participants also pointed out issues related to the way genomic research is currently conducted, particularly the inappropriate recruitment methods used for underrepresented populations, which often fail to consider their language and cultural needs (Quote 28, Participant #2). As previously mentioned, conducting scientific research with populations that have experienced social and racial prejudices requires more time and resources, which, according to several interviewees, is not commonly available in most genomic research (Quote 26–29, Participant #13–3). Many participants pointed to the high cost of genomic research, the lack of available technical tools, and the insufficient diversity in staff as examples of resource limitations that hinder research with underrepresented populations.

Finally, one participant explained that although DEI policies were initially intended to be beneficial, they are now causing more harm than good. According to this participant, these policies have become barriers to the participation of underrepresented populations, as they are fueling"tensions"and"racism"in society (Quote 30, Participant #6).

### Consequences and solutions to racial inequalities in genomic research

Table S3 [see Additional file 4] presents quotes from participants regarding the consequences of inequalities in genomic research and potential solutions. Nearly all participants noted that a key consequence of the lack of diversity in genomic research is an incomplete or inaccurate picture of the human genome (Quote 31, Participant #8), which impacts the treatment and diagnosis of diseases in underrepresented populations and those with rare diseases (Quote 32, Participant #13). Most participants mentioned that from a social perspective, the lack of diversity in genomics perpetuates existing social inequalities in society, creating a vicious circle (Quote 33, Participant #3). Some said that this negative feedback loop fuels the distrust and concerns of underrepresented communities regarding scientific research and the healthcare system. Finally, one participant working with Indigenous populations also noted that without a diverse reference group, the inability to diagnose rare diseases in underrepresented populations not only affects individuals but also their extended families and entire communities (Quote 34, Participant #13).

Regarding the solutions put forward by participants to diversify genomic research, the main idea expressed in most interviews was the need for more community involvement throughout the research process. Participants stressed that research projects should involve working with diverse communities and be designed by and for underrepresented populations (Quote 35, Participant #2). This means that all steps, from data collection to the governance of biobanks after the research, should be done by and for the communities participating in the project. For some participants, researchers can no longer rely on the traditional research approach of entering a community, collecting data, and then leaving (Quote 36). Several participants suggested involving community and/or organization representatives as intermediaries between the research team and the population to help engage with communities (Quote 37, Participant #9). As one participant stated, this approach must be paired with good communication and knowledge translation (Quote 38, Participant #12).

As mentioned, most participants pointed out the overall need for more resources and training for researchers. This means not only more funding and equipment to address gaps in genomics, but also the need for capacity building within communities to create a more diverse workforce in academia (Quote 39, Participant #13).

### Influences of DEI policies in genomic research

Finally, the influence of DEI policies on the participants'research projects was also discussed as a secondary topic during the interviews. Quotes from these discussions are available in Table 3. Firstly, some participants stated that DEI policies had little to no influence on their research work. This lack of influence had two dimensions. On the one hand, some researchers reported that these policies were unnecessary for their work, as recruitment was based on clinical characteristics (Quote 40, Participant #9). On the other hand, some participants attributed the lack of influence to their research being directly related to DEI topics. Finally, some participants felt that DEI policies had little impact because researchers could always find ways around them when applying for funding or carrying out a project. Therefore, the nature of the research remained unchanged (Quote 41, Participant #13).

**Table 3:** Influences of DEI policies in genomic research

Subtheme	Selected quotes
Lack of influence on research	Quote 40: *"For my research, because these were populations that were selected based on a diagnosis, so a brain tumor in the adults or pediatric cancer in children or rare inherited disorders, those were not selected based on equity, diversity or those kind of inclusion criteria. That's what was in front of us regardless of what your ethnicity was or your sexual orientation, and so. From a population recruitment standpoint, the population was a one-off defined by their disease."* Participant #9
	Quote 41: *"Close to not at all. So, there's a lot of, this is my perspective, but there's a lot of whistling towards trying to sort of look good and sort of like we feel good that we're addressing issues, but the reality is that it fundamentally [doesn’t] change what people are proposing to do. They're just managing the process to say what needs to be said in order to get the resources and the funding to do what they would want to do anyway. And so, I don't think it makes a profound difference in what happens in terms of research. We can certainly, you know, researchers are very good at managing application process to say what needs to be said to. Get the funding. But I don't think it changes the nature of the research that's pursued."* Participant #13
Positive influence on research	Quote 42: *"We have a whole working group looking at data diversity or inclusion. Not just the EDI aspect in the general project, but really in terms of data and then thinking about how we can first calculate metrics to find out what we have. Then think about how to support the generation of a more inclusive data set. I really think that this will bear fruit. I think, modifying existing projects to better capture these additional concepts. And also, I think, as I was saying before the question, even directly funding projects where this is the emphasis, I think that's very relevant and very positive."* Participant #4
	Quote 43: *"I've seen that carry through from not only the research institutes and the research funders, but also in the healthcare landscape and the health system decision makers, all of whom are now stakeholders of part of my equity research and that wouldn't have been possible, you know, before the pandemic or five years ago, now that the world has moved to prioritize equity, diversity and justice and access especially in the health space. It's become an impetus for my work to actually move forward."* Participant #5

However, several participants expressed more positive views of DEI policies (Quote 42, Participant #4). They noted that these new requirements promote ethnic and gender diversity in research that does not specifically target populations. Additionally, they encourage researchers who might not have otherwise included gender and ethnicity data in their studies. Some participants also shared that these policies increased their awareness of the importance of addressing inequities in research. Furthermore, DEI policies are expanding beyond the research community and are increasingly being adopted within the healthcare system, which is expected to positively impact underrepresented populations (Quote 43, Participant #5).

## Discussion

### Relation between categories, categorization and participation

Our exploration of the participation of underrepresented populations, categorization practices, and population descriptors reveals the complex nature of these issues. From our discussions, it became evident that participants viewed race as a social construct and as clearly part of a socio-historical construction. However, it was concerning that a few participants still maintained that race has a biological basis. This notion, which has its roots in colonialism and the eugenics and racial sciences of the nineteenth and twentieth centuries [35], still seems to persist in part in biomedical circles, a trend also observed in American-based studies of genomic researchers more than 12 years ago [23, 24]. While this was a minority view in our interviews, and participants acknowledged the problems with using race as a population descriptor in genomics, our findings highlight the continued persistence of racial thinking within the genomic field.

Nonetheless, we also observed important confusion and difficulty among participants in clearly distinguishing between race, ethnicity and ancestry, even though the categorization of genetic similarities using these population descriptors was part of their research. Similar findings were reported in American studies that assessed researchers’ perspectives on population categorization [21, 23–25, 27]. However, participants pointed out this confusion and complexity in categorizing populations stemmed from the non-discrete boundaries between groups. The mixing and diversity within human populations make it difficult to assign fixed and unchanging identities to a group. From an epistemological standpoint, it also highlights the socially constructed nature of these different categories [36]. For example, commonly used categories such as Black or Latino often fail to capture the full range of cultural and genetic diversity within these socially defined groups [23, 35]. As noted by Fatumo et al., of the 1.1% of African ancestry participants in GWAS studies worldwide, more than 90% of ethnolinguistic groups are still not represented [2]. Our study shows that the lack of harmonization in categorization practices is still present, even in the Canadian context.

Finally, our interviews highlighted the need to explore further the potential relationship between population descriptors and the participation of underrepresented populations in genomic research. Participants indicated a belief in such a relationship, though the direction of that association remained unclear. Above all, they emphasized the importance of properly contextualizing and communicating genomic research to underrepresented populations when assigning labels to study groups. This is especially important in light of the increasing “weaponization” of genetic and genomic research results by racist and far-right groups [37]. Non-discriminatory communication of data should, therefore, be a priority to prevent misuse and to avoid exacerbating racial discrimination towards specific communities. Furthermore, such communication could increase the participation of underrepresented populations in genomic research. More research is needed to determine the existence of such a relationship and its effects on participation.

### Mistrust and concerns: between geography, colonial legacy and research funding

Our study aimed to understand the reasons behind the low participation of underrepresented populations in genomic research from the perspectives of genomic researchers and explore solutions to increase their participation. Their views aligned with the scientific literature on the subject [10, 11]. As mentioned, participants noted that mistrust, concerns over data governance, and historical discrimination in scientific research could be the main drivers of the low participation of underrepresented populations in genomic research. Moreover, geographical factors, especially the divide between LMICs and HICs, were reported to play an important role in this lack of diversity. Since the most important genomic research institutions are in HICs, the research population will tend to reflect the demographic makeup of these regions. Similar views were also reported in Fatumo et al.’s paper on diversity in genomic studies [2]. We argue that this concentration of genomic research in a few HICs is the product of what Immanuel Wallerstein calls the world-system. According to Wallerstein, the European colonial project in the sixteenth century enabled the concentration of wealth, resources and knowledge in what he calls the core of the world-system (Western countries) by enforcing its scientific paradigm and socio-economical model to peripheral nations throughout the world, i.e. colonialism [38, 39]. The present geopolitical landscape is thus the legacy of the division between the core (HICs) and the periphery (LMICs) of the world-system [38, 39]. As a few participants noted, we argue that the lack of diversity in genomic research is deeply rooted in the legacy of this colonial division. Colonialism not only affects the global distribution of scientific funds, but also how underrepresented populations are treated in academia, healthcare, and society [40, 41]. In the Canadian context, Indigenous populations are particularly affected by the consequences of the colonial system [42]. In this sense, core-periphery dynamics are reflected within Canada, where Indigenous populations on the rural periphery continue to bear the consequences of an unequal relationship with the urban core, particularly in terms of access to social, economic, and health resources [42, 43]. In this context, the widespread mistrust and concerns within underrepresented populations can be understood as a lasting legacy of harmful colonial practices, which may still be perpetuated within our institutions. This is especially true for Indigenous populations in Canada, who often express a deep sense of distrust toward researchers, primarily due to unethical research practices historically carried out within their communities [15, 23, 43, 44]. However, broader research with underrepresented populations is still needed to fully understand the nature of their mistrust. It is important to note that to address the lack of trust within these populations, researchers and their institutions must make concerted efforts to build and demonstrate their trustworthiness to underrepresented groups whose lives may be impacted by the research [45]. We believe this is true in Canada and across the global research community.

### Tackling the issue: insights from researchers

Furthermore, our study suggests that genomic researchers are generally aware of the existing evidence on the causes of the underrepresentation of diverse populations. In addition to identifying these causes, our participants proposed several solutions aligned with the literature on the subject. Increased funding, capacity building, community-led work, data governance by communities, and education were among the solutions mentioned during our interviews, all featured in numerous studies focusing on the diversification of genomic studies [2, 15, 16]. A common theme among the proposed solutions to increase participation was the importance of actively engaging communities. This is particularly relevant in the Canadian context, where many underrepresented populations, such as Indigenous peoples, live in rural and remote areas with limited access to quality housing and healthcare resources [46]. In this regard, solutions require more than just a neutral stance toward the populations being studied; active engagement tailored to the specific needs and characteristics of these underrepresented groups is important for increasing their participation in research [47].

This perspective on passive versus active roles mirrors how participants viewed DEI policies. If the objectives of DEI policies in institutions are to diversify representation in both the workforce and research topics, our study participants highlight the need for more authentic and active integration of these policies. They emphasized that simply acknowledging the need for more diversity and inclusion is not enough; institutions must allocate time and resources to implement these policies. For instance, research involving Indigenous populations in Canada often demands more time and effort than research with the general population. This is because such research typically strives to develop co-constructed research projects with strong community engagement. In some cases, such measures are even mandatory, as required by ethics committees and the communities themselves [48]. However, research granting agencies in Canada typically provide funding for projects lasting no more than five years, which is often insufficient to build trust between researchers and Indigenous communities. As a result, Canadian funding policies may discourage researchers from pursuing long-term projects with Indigenous populations. To address this issue, these agencies could consider offering grants that extend beyond five years for research involving underrepresented populations.

As for researchers, we believe that genomic research with underrepresented populations would benefit from adopting a more multi-leveled approach, where social structures and individual/community needs are addressed simultaneously. Our vision for genomic research is rooted in the agentic paradigm proposed by Acolin and Fishman [49]. The authors advocate shifting population health research from a purely biomedical model to an agentic one that focuses on maximizing structural and individual resilience. This approach views health not as a result of causal determinism but as shaped by the interaction between individual aspirations and structural barriers or supports. [49]. In other words, rather than focusing on a single pathway to understand or address a health issue, we should aim for a broader perspective that considers the various factors involved. The complex nature of underrepresentation in genomic data calls for interventions and models that consider and address this complexity.

### Strengths and limitations

To our knowledge, this is the first Canadian-based study exploring categorization practices and researchers’ perspectives on the low participation of underrepresented populations in genomic research. Moreover, our qualitative approach provided a deeper understanding of the perceptions and opinions of the researchers than a quantitative design could have achieved, even though qualitative research typically does not aim to generalize findings [33]. However, our study may be subject to selection bias, as it is possible that participants who chose to take part in our research were already interested in issues related to underrepresentation. Additionally, due to the sensitive nature of our research topic, it is also possible that participants provided responses that were more socially acceptable, while researchers with differing opinions may have opted not to participate. A study utilizing a different methodology, such as an anonymized survey with probability sampling, could have provided a wider range of perspectives. Thus, our findings are specific to the time and context of the interviews.

Due to the time and workforce constraints of this study, which was part of a master’s thesis, we were unable to independently code the discussion transcripts with multiple team members. However, to mitigate this limitation, the research team collectively discussed the thematic analysis before approving the results. We also acknowledge that some of the population descriptors used in the interview guide were outdated in relation to current recommendations [20]. To avoid perpetuating this issue, we have limited the use of more controversial population descriptors in this article.

Finally, while the virtual format has limitations, particularly regarding access to computer tools, it allowed us to reach researchers in different cities across Canada without the need to travel. This format also proved useful for those not in remote areas, as it helped accommodate their busy schedules by removing travel constraints [50]. As a result, we were able to include researchers from nearly every major Canadian province involved in genomics (see Table 1). Given the exploratory nature of our study, an analysis of the association between participants’ demographics and their perspectives was not possible, but this should be explored in future research.

## Conclusion and implications for practice and research

Our study sheds light on the current views and attitudes of Canadian researchers regarding the low participation of underrepresented populations in genomic research. While most of our participants were aware of the current literature on the causes, consequences, and solutions to diversifying genomic research, there was significant confusion around the definitions and distinctions between race, ancestry, and ethnicity, which persisted when it came to population descriptors. This confusion suggests that challenges identified in earlier American studies still persist in the Canadian context [21, 23, 25]. More concerning, however, was that this confusion led some researchers to suggest a biological basis for race. Therefore, the confusion surrounding population categorization underscores the need for better education, consensus-building and harmonization of population descriptors, which would improve conceptual rigour and facilitate the transferability of results across genomic studies [51].

In this sense, genomic researchers in Canada would benefit from best practice guidelines, such as those from the American non-profit institution, the National Academies of Sciences, Engineering and Medicine (NASEM), on the use of population descriptors in genomic research [20]. To develop similar guidelines in Canada, a similar approach to NASEM should be prioritized, which includes convening experts from various fields. Future research should also involve consultation and co-production with the communities affected by these population descriptors, such as Indigenous populations. We also support the report's recommendations, notably on the need to clearly describe how race, ethnicity or ancestry are defined in each study and to collect a broader range of socio-demographic data rather than relying solely on ethnocultural origin. In addition, the inclusion of variables like structural racism, discrimination or other forms of oppression in biomedical research could also benefit genomic research [52]. For example, several studies assess exposure to structural racism and racial discrimination using a combination of factors like geographic segregation, socio-economic status, education, and body mass index [53]. Finally, we believe that a relationship may exist between population descriptors and the participation of underrepresented populations in genomic research. While more research is needed to confirm this, researchers need to prioritize effective communication, proper contextualization of research, and community involvement to ensure equity and inclusion throughout the research process [23, 35]. Nevertheless, the underrepresentation of diverse populations in genomics is not solely a concern for researchers. As we have outlined, it is a broader problem that intersects with social inequalities, the dynamics between HICs and LMICs, and the central role of funding agencies. Future research should engage a diverse range of stakeholders, beyond just researchers, to gain a deeper understanding of the issue and develop more effective solutions.

## Supplementary Information

Additional file 1.

Additional file 2.

Additional file 3.

Additional file 4.

Additional file 5.

Additional file 6.

## Data Availability

Interviews transcripts generated during the current study are not publicly available as per Ethics requirement that we maintain confidentiality of participants. We provided the interview guide in the Additional File 1.

## Abbreviations

DEI: Diversity, equity and inclusion
GWAS: Genome-wide association studies
HICs: High-income countries
LMICs: Low- and middle-income countries

## References

[1] National Human Genome Research Institute. The Cost of Sequencing a Human Genome 2021 [Available from: https://www.genome.gov/about-genomics/fact-sheets/Sequencing-Human-Genome-cost.

[2] Fatumo S, Chikowore T, Choudhury A, Ayub M, Martin AR, Kuchenbaecker K. A roadmap to increase diversity in genomic studies. Nat Med. 2022;28(2):243–50. 35145307 10.1038/s41591-021-01672-4 PMC7614889

[3] Martin A, Kanai M, Kamatani Y, Okada Y, Neale B, Daly M. Clinical use of current polygenic risk scores may exacerbate health disparities. Nat Genet. 2019;51:584–91. 30926966 10.1038/s41588-019-0379-x PMC6563838

[4] Mills MC, Rahal C. A scientometric review of genome-wide association studies. Communications Biology. 2019;2(1):9. 30623105 10.1038/s42003-018-0261-x PMC6323052

[5] Vyas DA, Eisenstein LG, Jones DS. Hidden in Plain Sight — Reconsidering the Use of Race Correction in Clinical Algorithms. N Engl J Med. 2020;383(9):874–82. 32853499 10.1056/NEJMms2004740

[6] Khoury MJ, Iademarco MF, Riley WT. Precision Public Health for the Era of Precision Medicine. Am J Prev Med. 2016;50(3):398–401. 26547538 10.1016/j.amepre.2015.08.031 PMC4915347

[7] Wojcik GL, Graff M, Nishimura KK, Tao R, Haessler J, Gignoux CR, et al. Genetic analyses of diverse populations improves discovery for complex traits. Nature. 2019;570(7762):514–8. 31217584 10.1038/s41586-019-1310-4 PMC6785182

[8] Manrai AK, Funke BH, Rehm HL, Olesen MS, Maron BA, Szolovits P, et al. Genetic Misdiagnoses and the Potential for Health Disparities. N Engl J Med. 2016;375(7):655–65. 27532831 10.1056/NEJMsa1507092 PMC5292722

[9] Ramraj C, Shahidi FV, Darity W Jr, Kawachi I, Zuberi D, Siddiqi A. Equally inequitable? A cross-national comparative study of racial health inequalities in the United States and Canada. Soc Sci Med. 2016;161:19–26. 27239704 10.1016/j.socscimed.2016.05.028

[10] Fisher ER, Pratt R, Esch R, Kocher M, Wilson K, Lee W, et al. The role of race and ethnicity in views toward and participation in genetic studies and precision medicine research in the United States: A systematic review of qualitative and quantitative studies. Mol Genet Genomic Med. 2020;8(2): e1099. 31867882 10.1002/mgg3.1099 PMC7005620

[11] Iltis AS, Rolf L, Yaeger L, Goodman MS, DuBois JM. Attitudes and beliefs regarding race-targeted genetic testing of Black people: A systematic review. J Genet Couns. 2023;32(2):435–61. 36644818 10.1002/jgc4.1653 PMC10349658

[12] Allen CG, Lenert L, Hunt K, Jackson A, Levin E, Clinton C, et al. Lessons Learned from the Pilot Phase of a Population-Wide Genomic Screening Program: Building the Base to Reach a Diverse Cohort of 100,000 Participants. J Pers Med. 2022;12(8). 10.3390/jpm12081228 PMC9410232 36013178

[13] Dolan DD, Pacia DM, Johnston J, Lee SS-J, Cho MK. Expanding the Agenda for a More Just Genomics. Hastings Center Report. 2024;54(S2):S2-S13. 10.1002/hast.4924 39707954

[14] Dolan DD, Cho MK, Lee SS-J. Spotlighting Structural Constraints on Decisions About Participation in Genomic and Precision Medicine. AJOB Empirical Bioethics. 2024;15(2):87–92. 10.1080/23294515.2024.2355893 PMC11180498 38776221

[15] Caron NR, Chongo M, Hudson M, Arbour L, Wasserman WW, Robertson S, et al. Indigenous Genomic Databases: Pragmatic Considerations and Cultural Contexts. Front Public Health. 2020;8:111. 32391301 10.3389/fpubh.2020.00111 PMC7193324

[16] Valiani AA. Frontiers of Bio-Decolonization: Indigenous Data Sovereignty as a Possible Model for Community-Based Participatory Genomic Health Research for Racialized Peoples in Postgenomic Canada. Genealogy. 2022;6(3):68.

[17] Washington V, Franklin JB, Huang ES, Mega JL, Abernethy AP. Diversity, Equity, and Inclusion in Clinical Research: A Path Toward Precision Health for Everyone. Clin Pharmacol Ther. 2023;113(3):575–84. 36423203 10.1002/cpt.2804

[18] Kalinoski ZT, Steele-Johnson D, Peyton EJ, Leas KA, Steinke J, Bowling NA. A meta-analytic evaluation of diversity training outcomes. J Organ Behav. 2013;34(8):1076–104.

[19] Government of Canada. Tri-Agency Statement on Equity, Diversity and Inclusion (EDI) 2022 [Available from: https://www.nserc-crsng.gc.ca/InterAgency-Interorganismes/EDI-EDI/index_eng.asp.

[20] National Academies of Sciences E, and Medicine. Using Population Descriptors in Genetics and Genomics Research: A New Framework for an Evolving Field. Washington, DC: The National Academies Press; 2023. 240 p. 36989389

[21] Bentz M, Saperstein A, Fullerton SM, Shim JK, Lee SS. Conflating race and ancestry: Tracing decision points about population descriptors over the precision medicine research life course. HGG Adv. 2024;5(1): 100243. 37771152 10.1016/j.xhgg.2023.100243 PMC10585473

[22] Lee SS. Racializing drug design: implications of pharmacogenomics for health disparities. Am J Public Health. 2005;95(12):2133–8. 16257939 10.2105/AJPH.2005.068676 PMC1449497

[23] Roberts DE. Fatal invention : how science, politics, and big business re-create race in the twenty-first century: New York : New Press, [2011] ©2011; 2011.

[24] Bliss C. Race Decoded: The Genomic Fight for Social Justice. Stanford: Stanford University Press; 2012. p. 280.

[25] Shim JK, Darling KW, Lappe MD, Thomson LK, Lee SS-J, Hiatt RA, et al. Homogeneity and heterogeneity as situational properties: Producing – and moving beyond? – race in post-genomic science. Social Studies of Science. 2014;44(4):579–99. 10.1177/0306312714531522 PMC4391627 25272613

[26] Popejoy AB, Crooks KR, Fullerton SM, Hindorff LA, Hooker GW, Koenig BA, et al. Clinical Genetics Lacks Standard Definitions and Protocols for the Collection and Use of Diversity Measures. Am J Hum Genet. 2020;107(1):72–82. 32504544 10.1016/j.ajhg.2020.05.005 PMC7332657

[27] Meloni M, Moll T, Issaka A, Kuzawa CW. A biosocial return to race? A cautionary view for the postgenomic era. Am J Hum Biol. 2022;34(7): e23742. 35275433 10.1002/ajhb.23742 PMC9286859

[28] Baribeau C, Royer C. L’entretien individuel en recherche qualitative : usages et modes de présentation dans la Revue des sciences de l’éducation. Revue des sciences de l’éducation. 2012;38(1):23–45.

[29] DeJonckheere M, Vaughn LM. Semistructured interviewing in primary care research: a balance of relationship and rigour. Fam Med Community Health. 2019;7(2): e000057. 32148704 10.1136/fmch-2018-000057 PMC6910737

[30] Malterud K, Siersma VD, Guassora AD. Sample Size in Qualitative Interview Studies: Guided by Information Power. Qual Health Res. 2016;26(13):1753–60. 26613970 10.1177/1049732315617444

[31] Guest G, Bunce A, Johnson L. How many interviews are enough? An experiment with data saturation and variability. Field Methods. 2006;18(1):59–82.

[32] Rampin R, Rampin V. Taguette: open-source qualitative data analysis. Journal of Open Source Software. 2021;6:3522.

[33] Paillé P, Mucchielli A, Ayotte H, Berger È, Céfaï D, Cournoyer L, et al., editors. L'analyse qualitative en sciences humaines et sociales2012.

[34] Corbin J, Strauss A. Basics of qualitative research: Techniques and procedures for developing grounded theory, 3rd ed. Basics of qualitative research: Techniques and procedures for developing grounded theory, 3rd ed. Thousand Oaks, CA, US: Sage Publications, Inc. p. xv, 379-xv, .

[35] Painter NI. The history of white people: WW Norton & Company; 2011.

[36] Lee SSJ. Excavating difference: Race in genomic medicine. 2018. p. 221–7.

[37] Carlson J, Henn BM, Al-Hindi DR, Ramachandran S. Counter the weaponization of genetics research by extremists. Nature. 2022;610(7932):444–7. 36261568 10.1038/d41586-022-03252-z

[38] Wallerstein I. World-Systems Analysis An Introduction: Duke University Press; 2004.

[39] Grosfoguel R. Epistemic Racism/Sexism, Westernized Universities and the Four Genocides/Epistemicides of the Long 16th Century. 2013:31–58.

[40] Amster EJ. The past, present and future of race and colonialism in medicine. CMAJ. 2022;194(20):E708–10. 35609910 10.1503/cmaj.212103 PMC9188792

[41] Hussain M, Sadigh M, Rastegar A, Sewankambo N. Colonization and decolonization of global health: which way forward? Glob Health Action. 2023;16(1):2186575. 36940174 10.1080/16549716.2023.2186575 PMC10035955

[42] Truth and Reconcilliation Commission of C. Honouring the truth, reconciling for the future : summary of the final report of the Truth and Reconcilliation Commission of Canada. 2015.

[43] Guillemin M, Gillam L, Barnard E, Stewart P, Walker H, Rosenthal D. “We’re checking them out”: Indigenous and non-Indigenous research participants’ accounts of deciding to be involved in research. International Journal for Equity in Health. 2016;15(1):8. 26772174 10.1186/s12939-016-0301-4 PMC4715344

[44] Riggan KA, Rousseau A, Halyard M, James SE, Kelly M, Phillips D, et al. “There’s not enough studies”: Views of black breast and ovarian cancer patients on research participation. Cancer Med. 2023;12(7):8767–76. 36647342 10.1002/cam4.5622 PMC10134334

[45] Graham M. Sharing genomic data for health research: institutional trust and trustworthiness, and informed consent. Can Med Assoc J. 2022;194(44):E1511. 36379558 10.1503/cmaj.221500 PMC9828933

[46] Canada S. 2021 Census of Population 2021.

[47] Gafari O, Bahrami-Hessari M, Norton J, Parmar R, Hudson M, Ndegwa L, et al. Building trust and increasing inclusion in public health research: co-produced strategies for engaging UK ethnic minority communities in research. Public Health. 2024;233:90–9. 38865828 10.1016/j.puhe.2024.05.007

[48] Government of Canada. TCPS 2 (2022) – Chapter 9: Research Involving the First Nations, Inuit, and Métis Peoples of Canada 2022 [Available from: https://ethics.gc.ca/eng/tcps2-eptc2_2022_chapter9-chapitre9.html.

[49] Acolin J, Fishman P. Beyond the biomedical, towards the agentic: A paradigm shift for population health science. Soc Sci Med. 2023;326: 115950. 37148746 10.1016/j.socscimed.2023.115950 PMC10154061

[50] Keen S, Lomeli-Rodriguez M, Joffe H. From Challenge to Opportunity: Virtual Qualitative Research During COVID-19 and Beyond. Int J Qual Methods. 2022;21:16094069221105076. 35692956 10.1177/16094069221105075 PMC9167989

[51] Jeske M, Saperstein A, Lee SS-J, Shim JK. Marginalized measures: The harmonization of diversity in precision medicine research. Social Studies of Science.0(0):03063127241288498. 10.1177/03063127241288498 39370888

[52] National Academies of Sciences E, and Medicine. Rethinking Race and Ethnicity in Biomedical Research. Wilson MR, Beachy SH, Schumm SN, editors. Washington, DC: The National Academies Press; 2024. 270 p.

[53] Dean LT, Thorpe RJ. What Structural Racism Is (or Is Not) and How to Measure It: Clarity for Public Health and Medical Researchers. Am J Epidemiol. 2022;191(9):1521–6. 35792088 10.1093/aje/kwac112 PMC9437815

