# Supplementary material for: Underrepresented populations in genomic research: a qualitative study of researchers’ perspectives
Source: BMC Med Genomics. 2025 Apr 16;18:72. doi: 10.1186/s12920-025-02140-5 (PMC12001558; doi:10.1186/s12920-025-02140-5)
Supplement: Supplementary file 1 — Additional file 1. [file 12920_2025_2140_MOESM1_ESM.docx]

**Semi-structured interview guide**

**Introduction**

Hello Dr or Prof. [name of researcher]. Thank you again for agreeing to participate in this research. As mentioned earlier, the goal of my research is to better understand the factors specific to the Canadian context that influence the participation of racialized people in genomics research from the perspective of researchers in the field. The expected duration is approximately 30 to 40 minutes and it will be audio-recorded. It consists of 12 open-ended questions as well as sub-questions. However, if you wish, it can be longer than the scheduled time. You don't have to answer every question if you're not comfortable and you can stop the interview at any time. Are these terms and conditions right for you?

**Topic 1: Categorization of population differences**

- In general, what populations does your genomics research focus on?
- What process do you use to differentiate and recruit populations in your studies?
  - What biological and/or social markers do you use to distinguish populations? (Self-identification or AIMs/admixture/clusters)
- How do you position yourself about the use of the concept of race in genomics research?
  - In your opinion, is there a distinction to be made between the concept of race and ethnicity?

If so, how do you distinguish between these two concepts?

- - There seems to be a debate in the scientific literature about the reintroduction of the concept of race in genomics. For some, it is an important conceptual tool to better include historically discriminated populations. However, for other, the heavy history behind the concept of race should force us to reject this concept in scientific practice. How do you position yourself about these two perspectives?

**Topic 2: Perspective on inequalities in genomics research between Caucasian and racialized populations**

- Research shows us that more than 80% of genomics studies are done in Caucasian populations. From your perspective, what could explain this lack of diversity in genomics research in Canada?
- What do you think are the consequences of this lack of diversity in genomics research? (At the health, social and scientific level)
  - In your opinion, what is the impact of this lack of diversity?

**Topic 3: Inclusion of racialized populations in studies**

- How do equity, diversity and inclusion policies in academic institutions and funding agencies influence your research?
  - What strategies are you implementing to address these inclusion imperatives?
- From your perspective, what would be the barriers and facilitators to the participation of racialized people in genomics research in Canada?
- In your opinion, does categorization of populations in terms of race and/or ethnicity hinder or promote the participation of racialized groups in genomics research? Please, explain your answer.

**Conclusion**

Do you have any other elements to add on an issue or theme that we did not address during this interview?

**Thank you very much for your generous responses and for the time given to this interview. Wishing you a good continuation in your research work.**
