# Supplementary material for: Underrepresented populations in genomic research: a qualitative study of researchers’ perspectives
Source: BMC Med Genomics. 2025 Apr 16;18:72. doi: 10.1186/s12920-025-02140-5 (PMC12001558; doi:10.1186/s12920-025-02140-5)
Supplement: Supplementary file 2 — Additional file 2. [file 12920_2025_2140_MOESM2_ESM.docx]

| **Table S1** Categorization and population descriptors | |
| --- | --- |
| **Subtheme** | **Selected quotes** |
| Population descriptors used | Quote 1: *"There are different variables that are sometimes included in the questionnaires of the different cohorts [...] not only the self-reported origin, race or ethnicity, but also all sorts of variables on the country of birth, the country of birth of each of these 4 grandparents that were collected.  Then there's also data on the religion of the individuals. So, by cross-referencing all these different types of data, we can sometimes gain a better understanding of individuals' origins."* Participant #10 |
| Perspectives on the concept of race | Quote 2: *"I don't like to see the word race. First of all, it's not a word I like very much. We know that race is something that really has more of a social history. It's a socio-historical concept, much more than a biological one."* Participant #6 |
|  | Quote 3: *"No, it's more... I'd say whether it's in French or English, it's more a matter of knowing exactly how to talk about these things. I find it much harder to know the right way to talk about these things than the basic scientific concepts. Because if you're wrong about the scientific definition of a word when you're talking about a scientific concept, it's much less significant than if you're wrong about the difficult history associated with these words. How do you use them?"* Participant #4 |
|  | Quote 4: *"I probably would lean towards race being a useful term for genomics. I don't think it should be rejected because it is...I think something that is relevant biologically and historically. I haven't thought deeply about it, because I don't necessarily do race based or ethnic based research, but I think those sorts of demographics are certainly at least a variable in the research. Whether it’s a relevant one, it might be up for debate, but there's definitely different genetics based on race, which are well known."* Participant #12 |
| Perspectives on the concept of ancestry | Quote 5: *"But if the intent is to, from the first school of thought or position, where the idea is to take a biological frame to an issue then it's imperative to understand that race and ethnicity is the social and ethnic cultural constructs, and that what they're looking at is ancestry."* Participant #5 |
|  | Quote 6: *"So [ancestry] for me is the combination, let's say, of the origins of all our ancestors in our tree that we go back in time, in generations, at a certain level. So, if you talk to me about my grandparents' ancestral origins, I know where my grandparents were born, for example. So, my [ancestry] will mainly reflect that of my grandparents or great-grandparents or let's say my known origins going back in time. So, for me, [ancestry] is the combination of the rather recent origins of my ancestors."* Participant #10 |
| Perspectives on the concept of ethnicity | Quote 7: *"Ethnicity, on the other hand, is also related to how individuals identify themselves, but ethnicity goes much further. In other words, there's much more fine-grained resolution, where individuals can identify themselves as, for example, French-Canadian from Quebec, etc. [...]Then again, it's also a social construct, ethnicity, which includes not only people's geographical origins, European, Asian...but also cultural variables like religion, culture, etc. Language is also part of it."* Participant #10 |
|  | Quote 8: *"Ethnicity, I would argue, is, you know, your ancestry, in other words, your genotype, which you can which we now can use to trace your origins, your geographic origins and cultural origins."* Participant #11 |
| Categorization practices | Quote 9: *"Typically, self-identification. So, we'll just ask people on surveys, do you identify as X. You know, what country were you born in? What country did you come from or did your parents come from? What racial group do you most identify with? So typically, it's through surveys."* Participant #3 |
|  | Quote 10: *"In each region, we ask that the 2 grandparents be from that region. In the case of the Acadian Peninsula, we'd like to have 2 grandparents who [were] born there, so it's really up to them to define whether they belong to that region[...]We ask in the questionnaires: do you have grandparents from this region? When they say no, [they are] basically excluded. Just what the participant reports."* Participant #8 |
|  | Quote 11: *" It's generally information on genetic markers. Then we often have information on the participants, but by looking at the genetic profile, we can correct or have more granularity on...Well, I'll give you an example. We have a recent paper, which was...we compared the immune response in European individuals vs. individuals [...] of African descent. Then we had information on both types of participants in our study. But after that, in our study, we measured quantitatively the percentage of admixture to finally, in certain cases, reclassify or make sure we were making the right comparisons."* Participant #4 |
|  | Quote 12: *"But obviously, my studies also use other factors to study population genetics. So, we obviously look at other sociological determinants that include health, age and sex at birth...And then we also look at geographical origins, i.e. where people live, to include this in our models. And then, if necessary, we'll include variables such as education, income."* Participant #10 |
| Importance of categorization | Quote 13: *"[...]we can't deny that there are segments of the population - well, just think of the indigenous populations, for example - that are more often the victims of all kinds of discrimination. And if we start saying well then we can't use the vocabulary of ethnicity, race, population groups at all, it's very difficult, because we'd practically have to rebuild new, more neutral words. The problem with the word race is not so much the concept. The fact that we have a concept to talk about how we refer to population groups socioculturally is not bad in itself. The problem is that the word race has been misused so much."* Participant #6 |
|  | Quote 14: *"I know that in my area of expertise, more specifically, understanding the role of variants in diseases and biology, we don't get away with it. It's really important to understand where the sample comes from, because it really has an impact on interpretation and our analyses. That's it. It's very true. It really does have an influence. But after that, there's all sorts of baggage around the words we use, around the implications and all that. It makes it a bit difficult."* Participant #4 |
| Limits of categories | Quote 15: *"I think the terms that the field uses are cultural terms that emerged in the 1950s and I think they don't mean much anymore. Arguably, most populations, at least in North America, are admixed to one or more degrees, and I don't know what it means to be, for example, Black or to be White or to be Asian. And if you look in each of those communities, the definition of those terms, it varies. What does it mean to be Asian? Well, if you're part of different Asian populations, it means different things. So, I think it's an often misused term in both the scientific literature and in the public domain. And I don't find it very valuable or useful."* Participant #11 |
|  | Quote 16: *"We can see this because we are currently working on the PRS across the different populations in Europe...we can see significant differences between Italy, Greece and the United Kingdom, and these are all people of European origin[...]When you make a map with the polymorphisms, with the thousands of polymorphisms we have, it's interesting because you can virtually redraw the distribution of Europe. So, you can see the Scandinavian countries, Italy and Spain. But these populations have a distinct [genetic] profile."* Participant #2 |
|  | Quote 17: *"...we have to be very careful that it doesn't become a substitute for the socio-economic determinants of health, in other words, instead of asking the real questions about why people from certain origins or certain [ancestries] are different from a genetic point of view, it remains that it could be because of other factors. And sometimes that obscures those other factors."* Participant #6 |
