# Supplementary material for: Underrepresented populations in genomic research: a qualitative study of researchers’ perspectives
Source: BMC Med Genomics. 2025 Apr 16;18:72. doi: 10.1186/s12920-025-02140-5 (PMC12001558; doi:10.1186/s12920-025-02140-5)
Supplement: Supplementary file 3 — Additional file 3. [file 12920_2025_2140_MOESM3_ESM.docx]

| **Table S2** Causes of underrepresentation of racialized people in genomic research | |
| --- | --- |
| **Subtheme** | **Selected quotes** |
| Geographical and demographic causes | Quote 22: *"So, I think there's probably a few factors. One would be funding. Historically, funding goes to or at least the bulk of funding goes to first world countries, not lower/middle income countries, so it’s easier to access and do this stuff on people that live within you know those regions."* Participant #12 |
|  | Quote 23: *"So obviously, if the funding is in North America or Europe, then African populations, where there is less funding, are not going to have easy access to it.  But even in Canada, urban or rural populations will have different barriers to participating in research. If you're invited to take part and you live 5 minutes from the research centre, it's much easier than if you live 2 hours from the research centre. So, these geographical barriers are very important, especially in the Canadian context."* Participant #10 |
|  | Quote 24: *"So, you tend to profile what's available to you. And so, I guess because most of the institutions that led the initial wave of genetic research around the world were based in predominantly white countries, then they kind of just profiled what was easiest for them to do."* Participant #7 |
| Feelings of fear and mistrust: Colonial past and social inequalities | Quote 25: *"[...] I think the barriers would be a lack of trust. So, I think there are some populations that have historically been exploited genomically and I think that's in Canada, but also worldwide. And I think there needs to be a long-term goal to build trust. That's not something that's going to happen overnight."* Participant #11 |
|  | Quote 26: *"But the big one I think for First Nations is because of historical bad practices in research, there is a lot of trepidation and concern and resistance to participating in research that is hard to address, and in most studies people do not have the resources or time to work their way through these challenges."* Participant #13 |
|  | Quote 27: *"There are also, of course, socio-economic barriers, which are major obstacles to access to genetic and genomic research. As we all know, it's hard to get access to specific clinical tests or clinical research, even if you come from a privileged or educated background."* Participant #10 |
| Causes linked to the field of genomics | Quote 28: *"So we can't approach the same question in the same way. Then you have to adapt from a research point of view, you have to adapt to the population you're communicating with. So, you can't have a single, universal message. It's the same in the field of personalised healthcare. If you want to be personalised, you're going to have to adapt your communication tools."* Participant #2 |
|  | Quote 29: *"From my experience that involves a lot of time at the beginning. So, going out into communities, speaking to community groups, explaining, taking the time to make connections and explain what your research is about and that takes more time then typically just handing out a flyer or sending out a survey. So, I think that that's one issue will be taking the time to build up trust in communities about research in general."* Participant #3 |
|  | Quote 30: *"At the risk of being controversial, I will put all the policies of what we call equity, diversity, and inclusion in the negative. I believe they started from good concepts and important values. But then it almost became nonsense. So, I think these policies cause deep damage. And I think that, for me, it’s a barrier; it’s a barrier to recruitment. It’s a barrier to regaining the trust of some of these groups. It also escalates tensions and racism, and so it does damage on that front as well. So, I think that the current policies—I’m not saying there aren’t any good ones—but there are some very bad ones, and it’s very problematic."* Participant #6 |
