# Supplementary material for: Underrepresented populations in genomic research: a qualitative study of researchers’ perspectives
Source: BMC Med Genomics. 2025 Apr 16;18:72. doi: 10.1186/s12920-025-02140-5 (PMC12001558; doi:10.1186/s12920-025-02140-5)
Supplement: Supplementary file 4 — Additional file 4. [file 12920_2025_2140_MOESM4_ESM.docx]

| **Table S3** Consequences and solutions to racial inequalities in genomic research | |
| --- | --- |
| **Subtheme** | **Selected quotes** |
| Consequences of the lack of diversity in genomic research | Quote 31: *"We don’t have genomic references for these ethnic groups. So, we may miss the interpretation of several variations that are unique to that population, which we may label as uncertain, even though it's a benign polymorphism that's common in the population. We can also miss pathogenic variations once again because they are not seen in Caucasians or people of European ancestry."* Participant #8 |
|  | Quote 32: *"[...]So, you have children who are left in what's called the diagnostic odyssey. And so, the diagnostic odyssey is this idea with very rare diseases, it's challenging to get a diagnosis. And if you don't have appropriate reference data, then you have a portion of people [...] that will not receive a successful diagnosis. Whereas if they were of European ancestry, you would be more likely to do so. [...]That has economic impacts, that has social impacts. It has long term planning impacts. And so, all of those pieces come together when you lack appropriate reference data for medical diagnosis."* Participant #13 |
|  | Quote 33: *"I think it almost becomes like a circular. Like, people will see that your research, nobody who participated in your research you know, was from my culture and my group, my race. And then they think well, that has nothing to do with me. And then I think it makes them less interested in research and then it kind of becomes a bit of a cycle."* Participant #3 |
|  | Quote 34: *"[...] they're a rare disease doesn't just affect the child, the rare disease affects not just the immediate family, but the extended family. And particularly in First Nations where you have very strong community engagement, it affects the broad community. And so, you have suffering that goes on and you have a lot of things that impacts everybody around those cases. And when we say rare diseases, rare diseases are individually rare but are collectively quite much more common."* Participant #13 |
| Diversifying genomic research through community involvement | Quote 35: *"So, the key issues for research institutes in Canada—and it must be written elsewhere too—are that it’s not just about working with First Nations, but as we say, it’s research by, for, and with First Nations. We need to work with them. Then it's up to them to decide what kind of biobank they want to build and what type of information they want to collect. They really need to take ownership of these data banks, this information, but we still have a long way to go."* Participant #2 |
|  | Quote 36: *"But it's it's the sense of partnership and the sense of humility. Beyond that, it's not this helicoptering of the scientists going into the communities taking what they need and getting out."* Participant #5 |
|  | Quote 37: *"And then utilizing community representatives who are trusted to help to convey those results to those communities what are the potential benefits of participation research? How might these be used? How have they been used or how why you personally benefit, or your family members personally benefit from it? Explaining all that information I think could be really helpful in improving participation in genomic research."* Participant #9 |
|  | Quote 38: *"Good communication, good involvement with communities, with good knowledge translation back to the community and not taking their data and running with it and becoming famous. Giving back to the communities that you know you are researching, maybe it's very useful information. I think that knowledge translation piece is probably severely lacking. [...] So that that would probably maybe be a good steppingstone is that engagement, knowledge translation and involvement of people that we're researching. As opposed to just, you know, taking their data and never hear from them again."* Participant #12 |
| Diversifying genomic research by providing resources and educating researchers | Quote 39: *"Second is you have to have a focus on capacity building. So, you need to be ready to invest to lift more people into the skill sets that are involved and that's takes, you know, 10 to 20 years to invest in that work, and so that that means that your commitment is not just to producing research papers in high impact publications. It's producing people as a much bigger weight in the system to make sure that your capacity building is highly valued in terms of the outputs of the research that you're doing."* Participant #13 |
